# Sensors and Biosensors for the Determination of Small Molecule Biological Toxins

**DOI:** 10.3390/s8096045

**Published:** 2008-09-26

**Authors:** Xiang-Hong Wang, Shuo Wang

**Affiliations:** 1 Key Laboratory of Food Nutrition and Safety, Ministry of Education, Tianjin University of Science and Technology, Tianjin 300222, P.R. China; 2 Faculty of Food Science and Technology, Agricultural University of Hebei, Baoding 071001, Hebei, P.R. China

**Keywords:** Review, sensors, small molecule biological toxin

## Abstract

The following review of sensors and biosensors focuses on the determination of commonly studied small molecule biological toxins, including mycotoxins and small molecule neurotoxins. Because of the high toxicity of small molecule toxins, an effective analysis technique for determining their toxicity is indispensable. Sensors and biosensors have emerged as sensitive and rapid techniques for toxicity analysis in the past decade. Several different sensors for the determination of mycotoxins and other small molecule neurotoxins have been reported in the literature, and many of these sensors such as tissue biosensors, enzyme sensors, optical immunosensors, electrochemical sensors, quartz crystal sensors, and surface plasmon resonance biosensors are reviewed in this paper. Sensors are a practical and convenient monitoring tool in the area of routine analysis, and their specificity, sensitivity, reproducibility and analysis stability should all be improved in future work. In addition, accuracy field portable sensing devices and multiplexing analysis devices will be important requirement for the future.

## 1. Introduction

Biological toxins are poisonous substances produced by living cells or organisms that are active at very low concentrations. Small molecule toxins are the most widely studied biological toxins, and usually include mycotoxins and small molecule neurotoxins.

Mycotoxins are a diverse group of organic compounds produced by fungal species, such as mushrooms, molds and yeast, commonly found in cereals and nuts. The negative health effects of mycotoxins are numerous: mycotoxins are carcinogenic, immunotoxic, nephrotoxic and teratogenic and are also known to be endocrine disruptors [1-3].

The neurotoxin saxitoxin is one of the most toxic non-protein substances known, and is produced by a number of marine algal species and contaminated shellfish. Saxitoxin was reported to cause neurotoxic effects, gastrointestinal symptoms and loss of memory [3]. Tetrodotoxin is a fish toxin which produces one of the most lethal intoxications caused by a marine species. The gonads, liver, intestines, and skin of the pufferfish can contain levels of tetrodotoxin sufficient to produce rapid and violent death [4, 5].

These biological toxins are responsible for food poisoning and have the potential to be used as biological warfare agents at the toxic dose. Different from other protein toxins, bacteria and viruses, small molecule toxins are all molecules of lower molecular weight, easy handled, poisonous at lower doses and existing in routine food samples. All of these features make them far harder to detect and defend against. Due to the poisonous nature of such small molecule toxins, an effective analysis technique for quantifying their toxicity is indispensable. The typical method widely used for the detection and quantification of biological small molecular toxins is high-performance liquid chromatography (HPLC) with UV and/or fluorescence detection (FLD) [6-8]. The latter provides a highly sensitive response, and this alternative has been widely used as a routine monitoring tool. The instrumental methods provide sensitive and specific assays but have the following problems:

1) They are very laborious, and not really suitable for screening large numbers of samples for fieldwork.
2) The extraction and clean-up processes involve numerous time-consuming steps.
3) Different derivatization reagents have been used for converting the toxins into the correspondent fluorescent derivatives, which is a complex analysis procedure and needs highly skilled personnel.

Rapid, sensitive and specific assay techniques are needed for the routine analysis/monitoring of food, water, and air samples for both natural and intentional contamination by these toxins. Sensors and biosensors have rapidly developed in the past decades because of their rapid, convenient and practicality. There have been several reviews about sensors and biosensors in the past two years; most of them are referring to a specific kind of sensor. Ricci *et al.* described the preparation, optimization and applications of Prussian Blue modified electrodes for sensors and biosensors [9]. In 2006, Andreescu *et al.* summarized the research performed during the past twenty years on cholinesterase biosensors [10]. There have been no reviews to date about sensors in the determination of small molecule biological toxins. This review extends the scope of sensors and biosensors in the determination of small molecule toxins; the advantages and disadvantages of each type of sensor are critically reviewed.

## 2. The mechanism of action of sensors and biosensors

### 2.1 General principles at work in detection by sensors

In the previous studies, the sensors used relied on the toxicological modes of action of toxins, for example, Cheun *et al.* employed a channel biosensor for the assay of paralytic shellfish poisons (PSP), as it was able to block Na^+^ channels [11, 12]. Campàs *et al.* developed an enzyme sensor for the electrochemical detection of the marine toxin okadaic acid, based on the inhibition of phosphatase by this toxin [13].

Antibodies can be generated and used in detection of small molecule toxins due to their inherent selectivity and sensitivity. Immunoassays are one of the most powerful methods for the detection and quantification of antigens and antibodies, in a much broader sense this also includes characterization methods for analyzing the immunological properties of analytes. Most of the immunoassays detecting small molecule toxins are based on competitive assays, and typical formats include the so-called direct, indirect, and sandwich ones. However, the exact choice is typically dependent up the particular application. Enzyme linked immunosorbent assays (ELISA) are generally used for sensor detection. Their signal transduction using colorimetric or chemiluminescent enzyme substrates, their incubation and wash steps are well suited to automated instruments.

### 2.2 Processing and classification of biosensors

Sensing can be defined as the use of recognition elements, most commonly biological in origin, for binding to a small toxin molecule. The binding event usually takes place using the specific binding of an antibody to a corresponding analyte [14-19], making them one of the most popular choices for the recognition element in many biosensors.

The binding event must then be transduced in a manner that signals the presence of the targeted analyte. Most immunoassay signal transduction mechanisms are optical, such as colorimetric, fluorescence, enhanced chemiluminescence, and optical fiber [15, 20-24]; other transduction mechanisms are also used in sensing procedures, for example: electrochemical [25-28, 13] and surface plasmon resonance [5, 29]. General, the sensor names contain the word “sensor” combined with another word indicating the transduction mechanism used.

Tissue biosensor, enzyme sensor and immunosensor were the commonly used sensors in the determination of biological small molecule toxins, with immunosensors being frequently used. Table 1 summarizes detailed and relevant information about the various immunosensorsused for the determination of biological small molecule toxins.

## 3. Different types of sensors and biosensors

### 3.1 Tissue biosensor

Cheun *et al.* developed a simple tissue biosensor for measuring Na^+^ channel blockers for the determination of tetrodotoxin (TTX) in 1996, and in 1998, using the same methods, more paralytic shellfish toxins (PSP) such as gonyautoxin (GTX) and saxitoxin (STX) have been detected. The tissue sensor response to each of the different PSP was recorded and the results compared with toxicities determined by the standard mouse bio-assay [11, 12].

Vangelis *et al.* and Siontorou, C. G. *et al.* explored the transduction of interactions of aflatoxin M_1_ with bilayer lipid membranes (BLMs). This can be used for the direct electrochemical sensing of aflatoxin M_1_ for the construction of single-use devices [25, 26].

### 3.2 Enzyme sensors

Enzyme sensors were widely used in the past two decades for the determination of organophosphorus insecticides [10, 30]. At present, enzyme also acts as labeled substance in the other biosensors. In 2007, Campàs *et al.* developed an enzyme sensor for the electrochemical detection of the marine toxin okadaic acid (OA) [13]. The strategy was based on the inhibition of the immobilized protein phosphatase by this toxin and the electrochemical measurement of the enzyme activity by the use of appropriate enzyme substrates, electrochemically activated after dephosphorylation by the enzyme. Colorimetric inhibition assays have demonstrated that the phosphatase from human red blood cells is more sensitive and provides a wider linear range than the one produced by genetic engineering. Two different enzyme substrates have been tested. These kinds of sensors described above rely on the inherent character of toxins, such as blocking of ion channels and inhibition of enzyme active. Since many toxins have similar toxicity mechanisms; these are better suited for qualitative analysis, and not suitable for accurate determination.

## 4. Immunosensors

### 4.1 Optical immunosensor

#### 4.1.1 Immunosensor based on optical waveguide lightmode spectroscopy technique

The optical waveguide lightmode spectroscopy (OWLS) technique was applied to the detection of aflatoxin and ochratoxin in both competitive and in direct immunoassays by Adányi *et al.* in 2007 [18]. After immobilizing the antibody or antigen conjugate for the direct or indirect measurement, respectively, the sensor chip was used in a flow-injection analyzer (FIA) system.

#### 4.1.2 Chemiluminescent immunosensor

Marquette developed a semi-automated membrane based chemiluminescent immunosensor integrated into a flow injection analysis system for the detection of the ‘diarrheic shellfish poisoning’ (DSP) toxin okadaic acid (OA) [22]. Anti-OA monoclonal antibodies were labeled with horseradish peroxidase for their use in a competitive assay, in which the free antigen of the sample competes with immobilized OA. Based on commercially available polyethersulfone membranes, this bioanalytical system exhibits a low non-specific binding of antibodies in the presence of mussel homogenate.

### 4.2 Electrochemical sensor

In 2002, Kreuzer *et al.* optimized a screen-printed electrode (SPE) system and developed an electrochemical immunosensor for seafood toxin analysis [27]. ELISA was primarily used to develop all toxin systems, prior to transferring to SPE. The SPE system is simple and cost-effective due to their disposable nature, and analysis time is complete within 30 min. In addition, analyses can be achieved outside of a laboratory environment allowing for in-field measurements. Recovery experiments on selected toxins using the relevant working ranges highlighted the functionality of these systems yielding a ±10% deviation for the true value.

There have been some reports on ochratoxin A (OTA) determination using electrochemical sensors. Alarcón *et al.* developed a monoclonal antibody based electrochemical immunosensor for the determination of OTA in wheat [16]. The assays were carried out using monoclonal antibodies in the direct and indirect format, thereby resulting in the development of disposable screen-printed electrodes for quantitative determination of ochratoxin A. Oliveira *et al.* studied the redox properties of OTA using electrochemical techniques which have the potential for providing insights into the biological redox reactions of this molecule. The *in situ* evaluation of the OTA interaction with DNA using a DNA-electrochemical biosensor is also reported in [31], and in 2008, Prieto-Simón *et al.* investigated two indirect competitive enzyme-linked immunosorbent assay (ELISA) strategies with different OTA immobilization procedures for the development of OTA electrochemical immunosensors. OTA levels in wine were detected by the immunosensor for validation [32]. In 2008, Khan *et al.* developed a sensitive CS/TiO_2_ bioactive electrode to measure OTA. The limit of detection was 10 ng/mL with a CS/TiO_2_ bio-electrode [33].

A new immunoassay concept for the determination of an aflatoxin B_1_ (AFB_1_)-based bio-electrocatalytic reaction on micro-comb electrodes was proposed by Liu *et al.* in 2006. The micro-comb electrode was fabricated by means of self-assembling horseradish peroxidase (HRP) and AFB_1_ antibody molecules onto gold nanoparticles (nanogold) to give functionalized biorecognition surfaces. The presence of nanogold provided a favorable microenvironment for the immobilized biomolecules and decreased the electron transfer impedance, leading to the direct electrochemical behavior of the immobilized HRP [34].

### 4.3 Surface Plasmon Resonance Biosensor

Mullett *et al.* developed a surface plasmon resonance (SPR) immunosensor to determine the concentrations of the mycotoxin fumonisin B_1_ (FB_1_) in spiked samples [29]. Polyclonal antibodies produced against FB_1_ were adsorbed onto a thin gold film substrate, which is coupled to a glass prism in the Kretschmann configuration. The output beam of a planar light-emitting diode is focused through the prism to excite SPR at the surface of the gold film.

Taylor *et al.* reported the quantitative antibody-based detection of tetrodotoxin (TTX) by an inhibition assay with a surface plasmon resonance (SPR) sensor. In their study, a novel anti-TTX antibody sensing surface was developed by chemically immobilizing TTX onto a gold film coated with a mixed self assembled monolayer consisting of amine terminated oligo-ethylene glycol (OEG) alkanethiols and hydroxyl terminated OEG alkanethiols. The ratio of amine to hydroxyl terminated OEG alkanethiols and TTX immobilization chemistry were optimized to maximize the specific anti-TTX binding, while minimizing non-specific binding. The calibration curves were reported for two antibody concentrations incubated with samples of varying TTX concentrations ranging from 0.01 to 10000ng/mL. The detection limit for TTX is defined as IC_20_ (20% inhibitory concentration), which is 0.3 ng/mL in this work. The corresponding calibration curve has a characteristic IC_50_ (50% inhibitory concentration) of 6 ng/mL [5].

Yu *et al.* investigated a simple biosensor comprising a polypyrrole film doped with chloride on a gold surface on a miniaturized surface plasmon resonance sensor for ochratoxin A detection. The SPR angle and the thickness of each film were monitored [35]. In 2005, the same authors synthesized a molecularly imprinted polypyrrole film on the surface plasmon resonance sensor instead of a gold surface, for detection of ochratoxin A. The molecularly imprinted polypyrrole film was electrochemically polymerized on the sensor surface from a solution of pyrrole and ochratoxin A in ethanol/water (1:9 v/v). The film growth was monitored *in situ* by gauging the increasing SPR angle. Binding properties of the molecularly imprinted polypyrrole film were investigated by loading ochratoxin A standard solutions into the integrated 20-μL flow cell [36].

Most of the literature described above concerns the development of sensors for the determination of toxins, but the practicality of these sensors in real analyses is questionable. In practice, toxins usually coexist in some complex matrices, multiplexing or simultaneous detection of multiple analytes is one of the most important prerequisites for small molecular toxins. Ligler *et al.* developed a portable array biosensor which used total internal reflection fluorescence excitation and planar waveguides patterned with capture antibodies to monitor for a wide variety of analytes, competitive immunoassays have been successfully developed for detection of small molecular toxins in the complex matrics such as food [20].

## 5. Conclusions

Some general conclusions of sensors and biosensors in the determination of small molecular toxins were presented below. Biosensing is distinct from other physiochemical methods, such as mass spectrometry, that can also be very sensitive and specific in their own right. It could be using as screening bio-tools for the assessment of the toxicity of a sample. In a large majority of all quoted studies, antibodies were used to a wide variety of targets because of their being specific to the target analyte.

The advantages of biosensing techniques compared with other traditional analysis techniques are summarized below:

1) Extraction and clean-up analytical steps were reduced, thereby shortening the process time, making it possible to monitor a large number of samples.
2) Separation and analysis procedure could be achieved at the same time, making it suitable for online automated analysis.
3) Neither high cost nor skilled personnel needed which make it very convenient to use.

Future research is expected to be useful in helping to overcome a number of shortcomings:

1) Specificity should be improved in order to help discriminate more efficiently between closely related toxins;
2) Sensitivity should be enhanced, enabling the detection of small amounts of target material within a high background matrix;
3) The device should be able to maintain binding even during repeated washing steps;
4) The stability of the sensors should be improved to allow long-term use.

Above all, small molecule toxins are ubiquitous in related food products and dangerous to handle because of their differing pathology and etiology and their potential presence in different materials and matrices. Sensors and biosensors provide a convenient screening tool for monitoring toxins during routine safety analyses. With the significant advances being made in science, future sensors and biosensors are expected to show a marked improvement in processing speed and efficiency, such that highly accurate portable sensing devices and multiplex analysis devices will be an important requirement for future biosensing platforms.

## Figures and Tables

**Table 1.** Immunosensors for the determination of small molecule biological toxins in the past decade.

**Type of sensor**	**Transducer or Mechanism of sensor**	**Analyte and detection limit**	**Reference**
Optical waveguide lightmode spectroscopy immunosensor	Interaction of antibodies and free antigen	Ochratoxin A 0.5 ng mL^−1^ Aflatoxin B1 10 ng mL^−1^	[18]
Chemiluminescent immunosensor	Interaction of antibodies and free antigen	Okadaic acid 0.2 μg /100g	[22]
Fluorometric biosensor	Immunoaffinity for specificity and fluorescence for a quantitative assay	Aflatoxins 0.1 ppb	[15]
Electrochemical sensor	Interactions of aflatoxin M_1_ with bilayer lipid membranes	Aflatoxin M_1_ 2 nM	[25]
Electrochemical immunosensor	Screen-printed electrode	Seafood toxin <1 ng mL^−1^	[27]
Electrochemical biosensors	Screen-printed electrode	Low-molecular weight compounds	[28]
Electrochemical enzyme sensor	Inhibition of immobilised protein phosphatase by toxin	Okadaic acid 22 mg L^-1^	[13]
Electrochemical immunosensor	Screen-printed electrodes	Ochratoxin A 0.35 ±0.04 μgL^−1^	[16]
Electrochemical sensor	Redox properties of OTA	Ochratoxin A 0.26 μM	[31]
Electrochemical immunosensors.	Screen-printed electrode	Ochratoxin A in wine 0.3 ng mL^-1^	[32]
Electrochemical sensor	Bio-electrocatalytic reaction on micro-comb electrode	Aflatoxin B_1_ 0.1 ng ml^-1^	[34]
Electrochemical sensor	Interactions of aflatoxin M_1_ with self-assembled metal-supported bilayer lipid membranes (s-BLMs)	Aflatoxin M_1_ 0.5 nM	[26]
Electrochemical sensor	CS/TiO_2_ bioactive electrode	Ochratoxin A 10 ng mL^-1^	[33]
Surface Plasmon Resonance Biosensor	Planar light-emitting diode	Fumonisin B_1_ 50 ng mL^-1^	[29]
Surface plasmon resonance	Interaction of antibodies and free antigen	Tetrodotoxin 0.3 ng mL^-1^	[5]
Surface plasmon resonance	Polypyrrole film doped with chloride on the gold surface	Ochratoxin A 0.1 μg mL^-1^	[35]
Surface plasmon resonance	Molecularly imprinted polypyrrole film on the surface	Ochratoxin A 0.05 ppm	[36]
Array biosensor	Reflection fluorescence excitation and planar waveguides	Deoxynivalenol 0.2 ng g^-1^ Ochratoxin A 0.8 ng g^-1^ Aflatoxin B1 0.3 ng g^-1^	[20]

## References

[1] Kiessling K.H., Pettersson H., Sandholm K., Olsen M. (1984). Metabolism of aflatoxin, ochratoxin, zearalenone, and three tricothecenes by intact rumen fluid, rumen protozoa, and rumen bacteria. Appl. Environ. Microb..

[2] JECFA (2002). Evaluation of Certain Mycotoxins in Food..

[3] D'Mello J.P.F. (2003). Food safety: contaminants and toxins..

[4] Matsumoto T., Nagashima Y., Kusuhara H., Ishizaki S., Shimakura K., Shiomi K. (2008). Pharmacokinetics of tetrodotoxin in puffer fish *Takifugu rubripes* by a single administration technique. Toxin.

[5] Taylor A.D., Ladd J., Etheridge S., Deeds J., Hall S., Jiang S. (2008). Quantitative detection of tetrodotoxin (TTX) by a surface Plasmon resonance (SPR) sensor. Sens. Actuat. B-Chem..

[6] Angelo V. (1998). Michelangelo, PDetermination of Zearalenone in corn by means of immunoaffinity clean-up and high-performance liquid chromatography with fluorescence detection. J. Chromatogr. A.

[7] Gonzalez-Penas E., Leache C., Viscarret M. (2004). Determination of ochratoxin A in wine using liquid-phase microextraction combined with liquid chromatography with fluorescence detection. J. Chromatogr. A.

[8] Kim E.-K., Maragos C.M., Kendra D. F. (2004). Liquid chromatographic determination of Fumonisins B_1_, B_2_, and B_3_ in corn silage. J. Agric. Food Chem..

[9] Ricci F., Palleschi G. (2005). Sensor and biosensor preparation, optimisation and applications of Prussian Blue modified electrodes. Biosens. Bioelectron..

[10] Andreescu S., Marty J.L. (2006). Twenty years research in cholinesterase biosensors: From basic research to practical applications. Biomol. Eng..

[11] Cheun B.S., Endo H., Hayashi T., Nagashima Y., Watanabe E. (1996). Development of an ultra high sensitive tissue biosensor for determination of swellfish poisoning, tetrodotoxin. Biosens. Bioelectron..

[12] Cheun B.S., Loughran M., Hayashi T., Nagashima Y., Watanabe E. (1998). Use of a channel biosensor for the assay of paralytic shellfish toxins. Toxicon.

[13] Campàs M., Marty J.L. (2007). Enzyme sensor for the electrochemical detection of the marine toxin okadaic acid. Anal. Chim. Acta.

[14] Mullett W., Lai E.P.C., Yeung J.M. (1998). Immunoassay of Fumonisins by a Surface Plasmon Resonance Biosensor. Anal. Biochem..

[15] Carlson M.A., Bargeron C.B., Benson R. C., Fraser A.B., Phillips T.E., Velky J.T., Groopman J.D., Strickland P.T., Ko H.W. (2000). An automated, handheld biosensor for aflatoxin. Biosens. Bioelectron..

[16] Alarcón S.H., Palleschi G., Compagnone D., Pascale M., Visconti A., Barna-Vetró I. (2006). Monoclonal antibody based electrochemical immunosensor for the determination of ochratoxin A in wheat. Talanta.

[17] Liu Y., Qin Z.H., Wu X.F., Jiang H. (2006). Immune-biosensor for aflatoxin B_1_ based bio-electrocatalytic reaction on micro-comb electrode. Biochem. Eng. J..

[18] Adányi N., Levkovets I.A., Rodriguez-Gil S., Ronald A., Váradi M., Szendrő I. (2007). Development of immunosensor based on OWLS technique for determining Aflatoxin B1 and Ochratoxin A. Biosens. Bioelectron..

[19] Prieto-Simón B., Campàs M., Marty J.L., Noguer T. (2008). Novel highly-performing immunosensor-based strategy for ochratoxin A detection in wine samples. Biosens. Bioelectron..

[20] Ligler F.S., Sapsford K.E., Golden J.P., Shriver-Lake L.C., Taitt C.R., Dyer M.A., Barone S., Myatt C.J. (2007). The array biosensor: portable, Automated systems. Anal. Sci..

[21] Dodeigne C., Thunus L., Lejeune R. (2000). Chemiluminescence as diagnostic tool. A review. Talanta.

[22] Marquette C.A., Coulet P.R., Blum L.J. (1999). Semi-automated membrane based chemiluminescent immunosensor for flow injection analysis of okadaic acid in mussels. Anal. Chim. Acta.

[23] Dower P.M., Farrell P.M., Gibson B.C. (2007). Optimal refractive index design for an optical fibre-based evanescent field sensor. Syst. Control Lett..

[24] Carter R., Jaeobs M., Lubrano M.B. (1997). Rapid detection of aflatoxin B1 with immunochemical optodes. Anal. Lett..

[25] Vangelis G.A., Dimitrios P.N. (1997). Electrochemical transduction of interactions of aflatoxin M_1_ with bilayer lipid membranes (BLMs) for the construction of one-shot sensors. Sens. Actuat. B-Chem..

[26] Siontorou C.G., Nikolelis D.P., Miernik A., Krull U.J. (1998). Rapid methods for detection of Aflatoxin M_1_ based on electrochemical transduction by self-assembled metal-supported bilayer lipid membranes (s-BLMs) and on interferences with transduction of DNA hybridization. Electrochim. Acta.

[27] Kreuzer M.P., Pravda M., O'Sullivan C.K., Guilbault G.G. (2002). Novel electrochemical immunosensors for seafood toxin analysis. Toxicon.

[28] Marrazza G., Chianella I., Mascini M. (1999). Disposable DNA electrochemical biosensors for environmental monitoring. Anal. Chim. Acta.

[29] Mullett W., Lai E.P.C., Yeung J.M. (1998). Immunoassay of Fumonisins by a Surface Plasmon Resonance Biosensor. Anal. Biochem..

[30] Palleschi G., Bernabei M., Cremisini C., Mascini M. (1992). Determination of organophosphorus insecticides with a choline electrochemical biosensor. Sens. Actuat. B-Chem..

[31] Oliveira S.C.B., Diculescu V.C., Palleschi G., Compagnone D., Oliveira-Brett A.M. (2007). Electrochemical oxidation of ochratoxin A at a glassy carbon electrode and *in situ* evaluation of the interaction with deoxyribonucleic acid using an electrochemical deoxyribonucleic acid-biosensor. Anal. Chim. Acta.

[32] Prieto-Simón B., Campàs M., Marty J.L., Noguer T. (2008). Novel highly-performing immunosensor-based strategy for ochratoxin A detection in wine samples. Biosens. Bioelectron..

[33] Khan R., Dhayal M. (2008). Nanocrystalline-bioactive TiO_2_-chitosan impedimetric immunosensor for ochratoxin A. Electrochem. Commun..

[34] Liu Y., Qin Z.H., Wu X.F., Hong J. (2006). Immune-biosensor for aflatoxin B_1_ based bio-electrocatalytic reaction on micro-comb electrode. Biochem. Eng. J..

[35] Yu J.C.C., Lai E.P.C. (2004). Polypyrrole film on miniaturized surface plasmon resonance sensor for ochratoxin A detection. Synth. Met..

[36] Yu J.C.C., Lai E.P.C. (2005). Interaction of ochratoxin A with molecularly imprinted polypyrrole film on surface plasmon resonance sensor. React. Funct. Polym..

